# Bowel Blockage Without a Block: Amyloidosis Presenting as Chronic Intestinal Pseudo-Obstruction

**DOI:** 10.7759/cureus.84189

**Published:** 2025-05-15

**Authors:** Rangesh Modi, Guy Nguefang, Freny Patel, Prince Modi, Edgar M Luna Landa

**Affiliations:** 1 Gastroenterology and Hepatology, The University of Chicago Medicine, Chicago, USA; 2 Internal Medicine, Texas Tech University Health Sciences Center, Odessa, USA; 3 Internal Medicine, Pramukhswami Medical College, Karamsad, IND

**Keywords:** chronic intestinal pseudo obstruction (cipo), dysmotility, gi amyloidosis, prucalopride, small bowel disease

## Abstract

We present the case of a 61-year-old man with a history of schizophrenia and non-ischemic cardiomyopathy who was admitted with chronic nausea, vomiting, and abdominal pain. His clinical course was marked by recurrent hospitalizations due to persistently dilated small bowel and multiple exploratory laparotomies, all failing to yield a definitive diagnosis, raising suspicion for chronic intestinal pseudo-obstruction. Extensive testing for vascular, paraneoplastic, infectious, and autoimmune causes was unremarkable. Given his unexplained cardiomyopathy and elevated serum light chains with a mild M spike, amyloidosis was suspected. A biopsy of the abdominal fat pad with Congo red staining confirmed amyloid deposition. His symptoms showed partial improvement with prucalopride, but he continues to require total parenteral nutrition and a venting gastrostomy tube for symptom management. Amyloid subtyping and a bone marrow biopsy are pending to determine the underlying etiology.

## Introduction

Chronic intestinal pseudo-obstruction (CIPO) is a rare and debilitating gastrointestinal (GI) motility disorder that mimics mechanical bowel obstruction but lacks any anatomical occlusion [1]. While CIPO can be idiopathic or secondary to a variety of systemic conditions, amyloidosis is an infrequent yet important etiology, and patients often develop intestinal failure needing total parenteral nutrition (TPN) [1-3]. GI amyloidosis can have varying symptoms, such as bleeding, pain, and dysmotility, based on the type of amyloid and depth of tissue involvement [4]. The small bowel is frequently involved in all subtypes of GI amyloidosis [5,6]. While imaging and manometry findings are nonspecific in small bowel amyloid [7,8], the endoscopic findings can vary from polyp-like protrusions to mucosal thickening or granularity [9]. However, in small bowel amyloid-causing CIPO, the macroscopic appearance on endoscopy may be normal, as deeper neuronal or smooth muscle tissue layers are involved that cannot be assessed on a superficial endoscopic biopsy [9]. Hence, an abdominal fat pad or other involved solid organ biopsy may be needed for diagnosis [9,10]. Treatment is largely supportive, and treating the underlying etiology may or may not resolve amyloid-associated CIPO, foreshadowing poor prognosis [10].

This case underscores the diagnostic complexity of secondary CIPO and highlights the importance of maintaining a high index of suspicion for infiltrative processes such as amyloidosis, especially in the setting of unexplained cardiac dysfunction and elevated plasma light chains. The eventual diagnosis of GI amyloidosis was confirmed through abdominal fat pad biopsy after failure of conventional imaging and endoscopic evaluation. Notably, the patient's symptoms improved with prucalopride, a selective 5-HT4 agonist, suggesting a potential role for this agent in amyloid-associated dysmotility.

## Case presentation

A 61-year-old African-American man with a history of schizophrenia and non-ischemic cardiomyopathy (ejection fraction 20%) presented to the emergency department with several weeks of nausea, non-bloody vomiting, and generalized abdominal pain. Over the previous four months, he had multiple hospital admissions for similar symptoms. During those admissions, abdominal CT scans consistently showed segmental small bowel dilatation without a definitive transition point, and he underwent two exploratory laparotomies, both of which revealed dilated small bowel loops without mechanical obstruction. He had no history of prior abdominal surgeries and no relevant family history, and he denied smoking, alcohol use, non-steroidal anti-inflammatory drug (NSAID) use, or herbal supplements. His chronic medications included clozapine 100 mg daily and quetiapine 200 mg at bedtime, both of which were held on admission due to the risk of worsening GI motility. On presentation, vital signs were stable: blood pressure 112/68 mmHg, heart rate 80 bpm, respiratory rate 16 breaths/min. He was afebrile and saturating at 94% on room air. The physical exam was notable for a distended abdomen with diffuse tenderness and decreased bowel sounds.

Laboratory evaluation revealed normocytic anemia with otherwise normal white blood cell count, differential, and platelet count. Metabolic and renal function profiles showed mild hypercalcemia, elevated blood urea nitrogen (BUN), and metabolic alkalosis, indicating dehydration from upper GI losses (vomiting). The remainder of the electrolytes and glucose were within the reference range. Liver function testing was normal, and the patient had hypoproteinemia, as evidenced by low total protein and albumin. Serum C-reactive protein was elevated, and lactate was normal. Serum thyroid-stimulating hormone (TSH) and cortisol were within normal range. Urinalysis was unremarkable. Serum B12, folate, and thiamine were in the normal range. Immunoglobulin testing showed an elevated IgG with otherwise normal IgG4, IgA, and IgM. Serum kappa and lambda-free light chains were elevated with an elevated kappa/lambda ratio. Serum protein electrophoresis revealed a small M spike of 0.3 g/dL in the gamma globulin region. Urine protein electrophoresis showed no monoclonal proteins. These findings were suspicious for monoclonal gammopathy of unknown significance or a smoldering myeloma. Table 1 shows detailed laboratory values with normal reference ranges.

**Table 1:** Detailed initial laboratory values with reference range. SI: Système International; CBC: complete blood count; WBC: white blood cell; g: gram; L: liter; dL: deciliter; mmol: millimole; nmol: nanomole; ml: milliliter; µL: microliter; mg: milligram; pg: picogram; ng: nanogram; mcg: microgram; mEq: milliequivalent; U: units; uIU: international units; BUN: blood urea nitrogen; AST: aspartate aminotransferase; ALT: alanine aminotransferase; ALP: alkaline phosphatase; TB: total bilirubin; TP: total protein; CRP: C-reactive protein; TSH: thyroid stimulating hormone; Ig: immunoglobulin

Laboratory type	Test (Blood)	Measured value	Reference range in SI units
CBC	WBC	9 x10³/µL	3.5-11 x10³/µL
	Hemoglobin	8.9 g/dL	13.5-17.5 g/dL
	Platelet	202 x10³/µL	150-450 x10³/µL
Metabolic profile	Glucose	62 mg/dL	60-99 mg/dL
	Sodium	142 mmol/L	135-145 mmol/L
	Potassium	3.7 mEq/L	3.5-5 mEq/L
	Chloride	102 mmol/L	98-108 mmol/L
	Bicarbonate	33 mmol/L	23-30 mmol/L
	Magnesium	1.8 mg/dL	1.6-2.5 mg/dL
	Corrected calcium	10.3 mg/dL	8.4-10.2 mg/dL
	Phosphate	2.8 mg/dL	2.5-4.4 mg/dL
Renal function	BUN	30 mg/dL	7-20 mg/dL
	Creatinine	0.84 mg/dL	0.5-1.4 mg/dL
Liver function	AST	23 U/L	8-37 U/L
	ALT	8 U/L	8-35 U/L
	ALP	58 U/L	50-150 U/L
	TB	0.3 mg/dL	0.1-1 mg/dL
	TP	5.3 g/dL	6-8.3 g/dL
	Albumin	2.2 g/dL	3.5-5 g/dL
Miscellaneous	CRP	20 mg/L	<5 mg/L
	Lactate	0.6 mmol/L	0.5-2 mmol/L
Endocrine	TSH	2.3 uIU/ml	0.3-4 uIU/ml
	Cortisol	14.4 mcg/dL	6-18.4 mcg/dL
Vitamins	B12	800 pg/ml	240-900 pg/ml
	Folate	14.5 ng/ml	4-26 ng/ml
	Thiamine	154 nmol/L	70-180 nmol/L
Immunologic	IgG	1763 mg/dL	800-1700 mg/dL
	IgA	447 mg/dL	100-490 mg/dL
	IgG4	61.2 mg/dL	2.4-121 mg/dL
	IgM	66 mg/dL	50-320 mg/dL
	Kappa light chain	6.6 mg/dL	0.33-1.94 mg/dL
	Lambda light chain	3.89 mg/dL	0.57-2.63 mg/dL
	Kappa/Lambda ratio	1.7	0.26-1.65

Infectious workup, including *Trypanosoma cruzi *antibody, HIV, hepatitis panel, syphilis serology, and stool polymerase chain reaction (PCR) for bacterial and viral pathogens (including *C. difficile*), was negative. Autoimmune testing revealed a positive antinuclear antibody (ANA) with a nucleolar pattern at a titer of 1:2560. However, other serologic markers, including anti-Scl-70, centromere, dsDNA, SSA/SSB, p-ANCA, c-ANCA, RNA polymerase III, and myositis antibodies, were negative. Serum autoimmune and paraneoplastic GI dysmotility antibody panel was negative (AchR ganglionic neuronal antibody, ANNA-1, AP3B2, CASPR-2 IgG, DPPX, LGI1-IgG, PCA-2).

**Figure 1:**
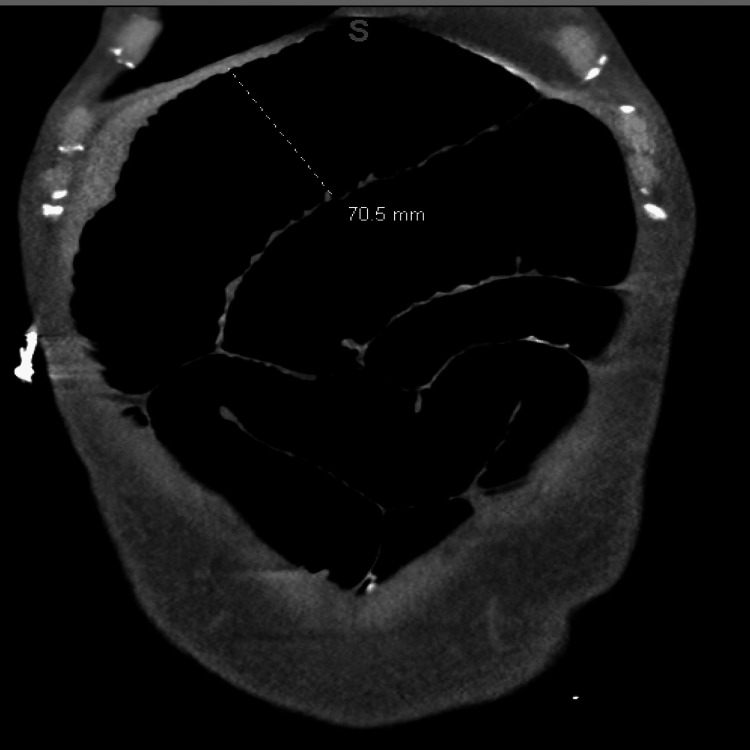
CT scan of the abdomen and pelvis with contrast showing dilated small bowel with no transition point up to 7 cm.

**Figure 2:**
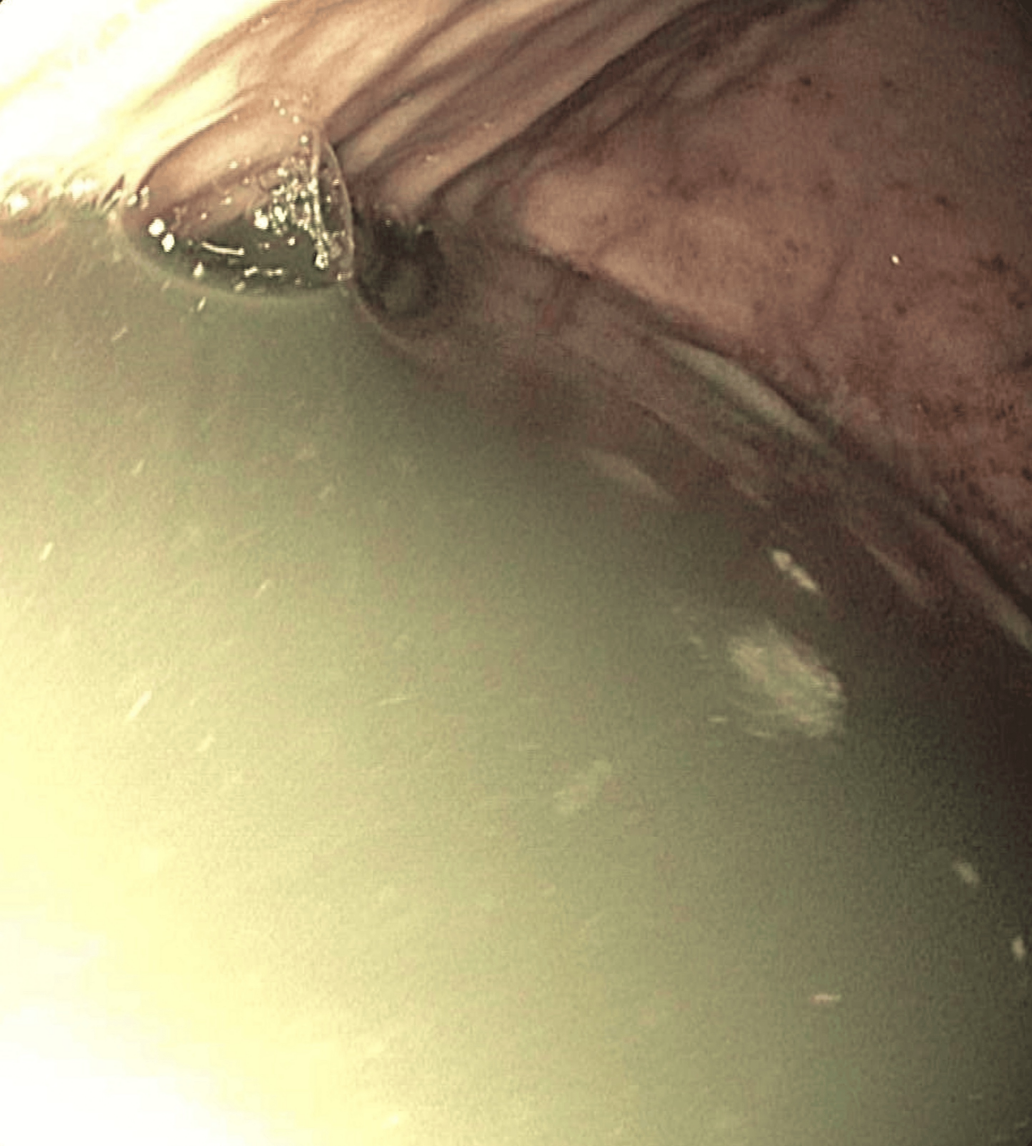
Upper endoscopy showing a fluid-filled stomach due to chronic intestinal pseudo-obstruction.

**Figure 3:**
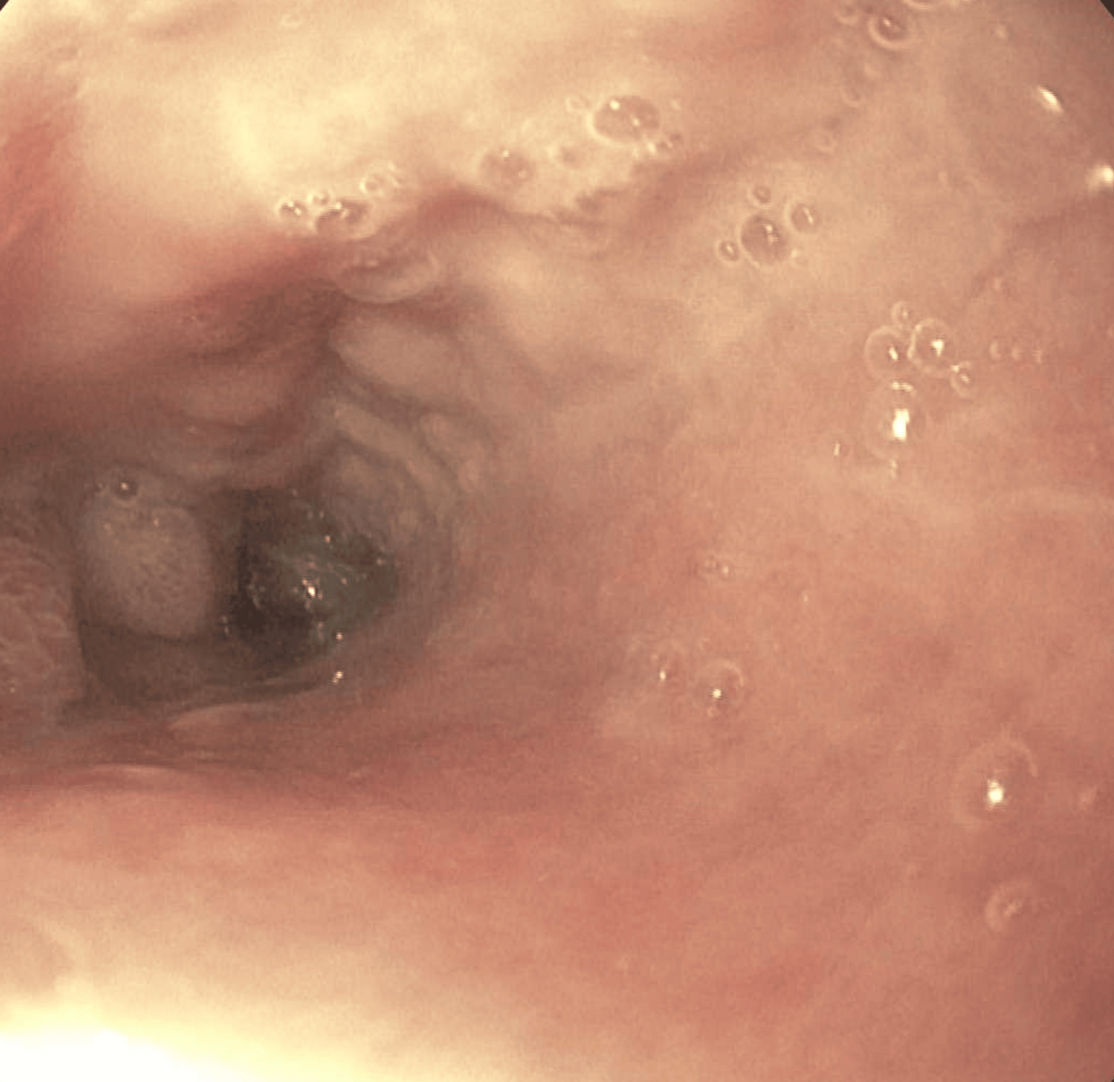
Upper endoscopy showing dilated duodenum with distal narrowing.

Imaging included a CT of the abdomen and pelvis showing duodenal and proximal jejunal dilatation up to 7 cm (Figure 1), with tapering and decompressed distal ileum. CT angiography ruled out mesenteric ischemia or vascular stenosis. CT of the chest was unremarkable, aside from cardiomegaly. MRI of the brain and spine showed no abnormalities. A small bowel follow-through demonstrated multiple dilated loops with sluggish peristalsis; contrast reached the colon within two hours. Upper endoscopy revealed a normal esophagus and fluid-filled stomach (Figure 2), with a diffusely dilated duodenum and a distal narrowing that could not be traversed (Figure 3); biopsies taken from the stomach were negative for amyloid on Congo red staining. Given persistent symptoms and lack of improvement despite cessation of clozapine and quetiapine for over six weeks, the clinical picture suggested CIPO. Rheumatology evaluation found no clinical features of scleroderma or other systemic autoimmune disease. Neurologic examination and spinal imaging were unremarkable.

**Figure 4:**
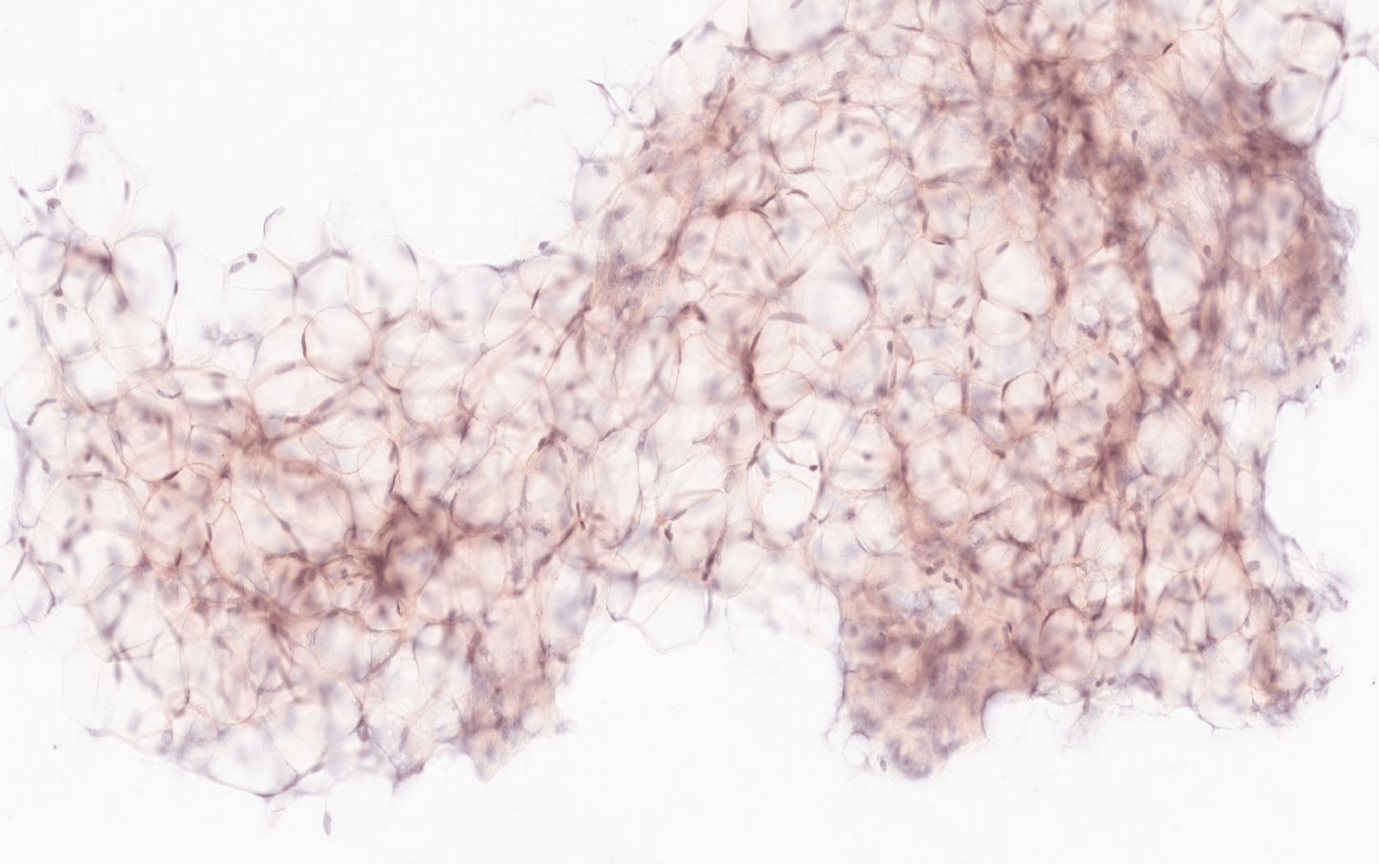
Abdominal fat pad biopsy showing reddish pink positive Congo red staining for amyloid material.

Due to ongoing high nasogastric output and failure of conservative measures, including bowel rest, TPN, and nasojejunal decompression, a diagnostic laparoscopy was performed. It revealed alternating pale, decompressed small bowel segments and dilated, pink, peristaltic loops without evidence of mechanical obstruction or significant adhesions. The ileocecal valve and colon appeared normal. A laparoscopic venting gastrostomy tube was placed, but a full-thickness bowel biopsy was not obtained. However, an abdominal fat pad biopsy demonstrated Congo red-positive deposits (Figure 4), confirming the diagnosis of amyloidosis. Amyloid subtyping and bone marrow biopsy are pending to determine the underlying etiology, which could be light chain amyloid given his elevated plasma light chains and M spike. Cardiac MRI showed pericardial effusion and diffuse patchy late gadolinium enhancement involving basal segments of the left ventricle and the right and left atrium, highly suggestive of cardiac amyloidosis.

**Figure 5:**
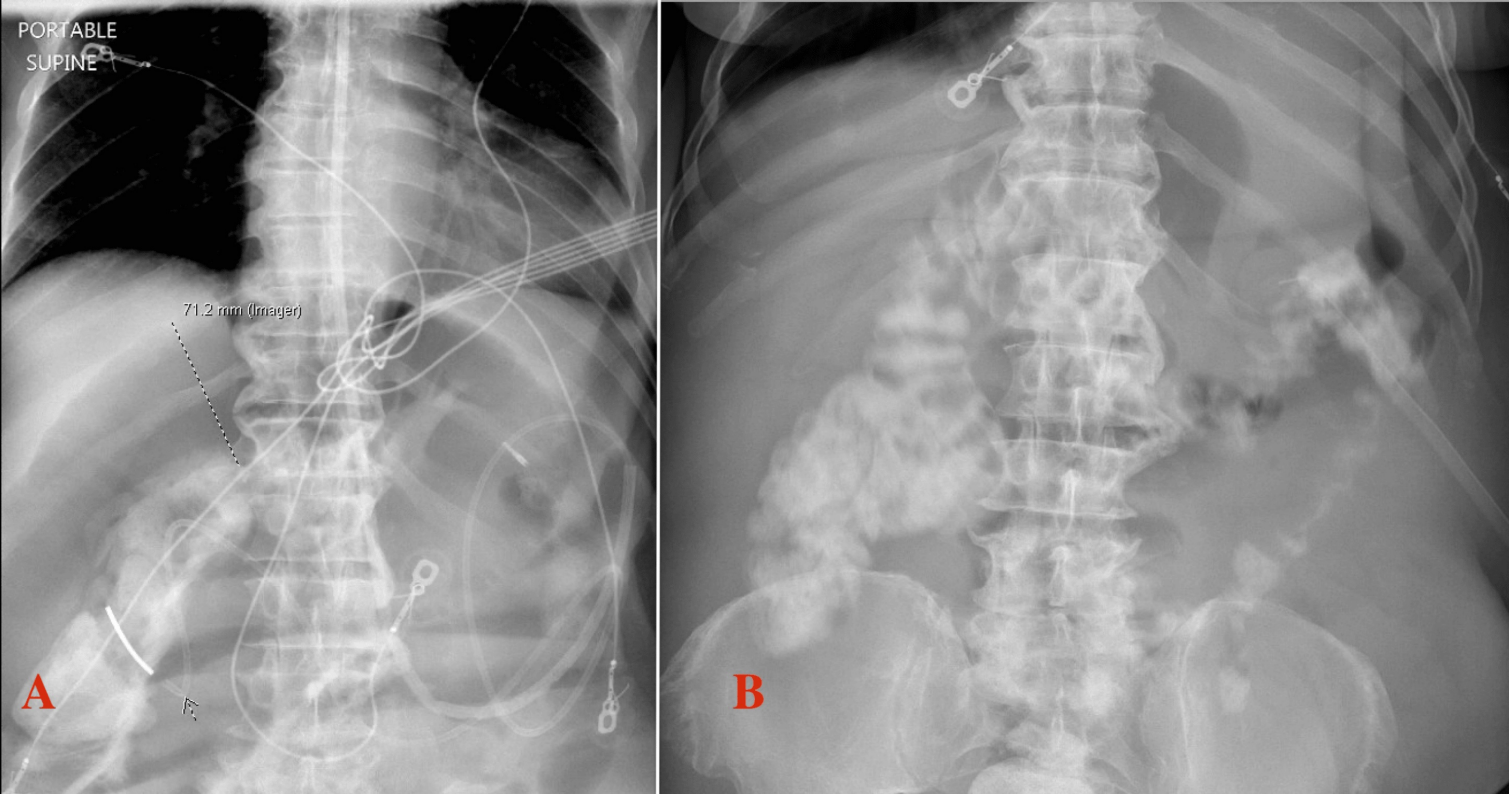
Abdominal X-ray. Abdominal X-ray showing small bowel dilation before (A) and after prucalopride (B). Nasojejunal tube is seen in (A) and percutaneous gastrostomy tube in (B).

He had previously received erythromycin with no effect, and metoclopramide was avoided, given his underlying psychiatric illness. He was started on prucalopride 2 mg daily with improvement in small bowel dilation on abdominal X-ray after five days of therapy. His gastrostomy tube outputs reduced from 2.5 L to 800 mL/day. Figure 5 depicts the improvement in small bowel dilation before and after prucalopride.

He continues to be on TPN and gastric decompression due to persistent abdominal cramping and nausea. We plan to start an oral diet when his GI symptoms reduce and he can tolerate a gastrostomy clamp trial.

## Discussion

CIPO is a severe GI motility disorder characterized by persistent symptoms of bowel obstruction without radiologic, surgical, or endoscopic evidence of a mechanical cause [1]. It accounts for approximately 9.7% of adult chronic intestinal failure cases [2]. The term intestinal pseudo-obstruction was introduced in 1958 by Dudley et al. in a case series of 13 patients with intestinal obstruction without evidence of mechanical process or lesion [3]. Eventually, the term CIPO has served as an umbrella term for many disorders causing small bowel or generalized gastrointestinal dysmotility (GID) in the absence of a mechanical obstruction [3]. Primary CIPO is due to genetic neuropathies or myopathies, typically starting in childhood [3]. While some cases remain idiopathic, secondary causes include infections (Chagas, cytomegalovirus, Epstein-Barr virus), connective tissue disorders (lupus, dermato or polymyositis, scleroderma), neuromuscular disorders (myasthenia gravis, autoimmune myositis or ganglionitis, Guillain-Barre), hormonal disorders (hypothyroidism), drug-induced disorders (narcotics, anticholinergics), paraneoplastic disorders (thymoma, lung, breast, lymphoma), radiation, and miscellaneous disorders (Ehlers-Danlos, amyloidosis) [3]. Management depends on the underlying etiology, such as treating infection or withdrawing offending drugs, but most cases remain unresolved. In cases with debilitating GI symptoms despite symptomatic management (prokinetics, antiemetics), TPN is used to address nutritional needs [3].

According to a retrospective study involving 19 patients with amyloid light-chain (AL) amyloidosis, the GI tract was the fourth most common organ involved after the kidney, bone marrow, and heart [4]. GI amyloid can cause a variety of symptoms, such as abdominal pain, spontaneous bowel perforation, weight loss, dysmotility (pseudo-obstruction), and GI bleeding [4]. In the GI tract, amyloid deposition frequently involves the small bowel [5]. A recent systematic review reported that patients with amyloidosis presenting with GID had a mean age of 62.1 years, and approximately 47.4% exhibited partial or complete pseudo-obstruction [5]. The majority of cases were from AL or amyloid A (AA amyloidosis) [5]. However, transthyretin amyloidosis (ATTR) can also involve the small bowel and cause CIPO [6]. The pathophysiology of CIPO in small bowel amyloid is due to amyloid infiltration between the muscle fibers of the intestinal smooth muscle, causing pressure atrophy of the adjacent fibers [3]. The involvement of the myenteric plexus by amyloid deposition and vascular insufficiency also results in hypomotility of the affected intestine [3].

The CT scan findings for small bowel amyloidosis are nonspecific and show dilated or thickened small bowel loops. Radiographic non-augmented oral barium or water-soluble contrast small bowel-follow-through study shows small bowel anatomy and an estimated transit time of contrast reaching the colon from the time of ingestion, serving as a useful diagnostic tool [7]. Normal transit time ranges from 30 to 120 minutes [7]. Small bowel amyloid-causing CIPO shows delayed transit and multifocal bowel dilation [8]. Small bowel manometry can be used to diagnose myopathic or neuropathic patterns of alterations in CIPO [8]. However, only a limited number of centers perform this procedure, and the results may be confounded by the use of medications slowing gut motility (anticholinergic or opiates) [8]. Hence, these may not provide a specific diagnosis but merely a hint into the pathophysiology of underlying dysmotility. In cases of amyloidosis, small bowel manometry shows a myopathic pattern of low-amplitude contractions (<20 mmHg) at affected sites [8]. Endoscopic findings can vary depending on the type of amyloidosis: polypoid lesions, mucosal protrusions, and thickened valvulae conniventes are more frequently associated with AL amyloidosis, whereas AA amyloidosis typically causes diffuse mucosal friability and ulcerations [9-11]. 

Histologically, amyloid appears homogeneous and amorphous under light microscopy [8]. It stains pink with hematoxylin and eosin and displays metachromasia with methyl violet [8]. Congo red is the most specific stain, which produces the characteristic red appearance in normal light and apple-green birefringence in polarized light [8]. However, diagnostic yield in small bowel biopsy in AL amyloid can be low, and many cases require biopsy from other involved organs, such as the kidney or abdominal fat pad [12].

Once the diagnosis of amyloidosis is confirmed, determining the subtype is essential, as it guides treatment by addressing the underlying disorder responsible for elevated amyloid precursors [13]. Prognosis depends on the type of amyloid involved; patients presenting with pseudo-obstruction generally have a poor prognosis [13]. The management of GI amyloidosis presenting with CIPO is challenging and largely supportive. Treatment strategies include dietary modifications, adequate hydration, and prokinetic agents such as erythromycin or prucalopride to promote motility [13]. Parenteral nutrition is indicated in severe cases of chronic GID associated with malnutrition [13]. Targeted therapy, such as chemotherapy and stem cell transplantation for AL amyloidosis and treatment of the underlying inflammatory disorder in AA amyloidosis, may ameliorate symptoms [13].

Our case highlights the methodological assessment for small bowel dysmotility of unknown etiology and identifying multi-system organ involvement clues, such as unexplained non-ischemic cardiomyopathy in our case. The index of suspicion for amyloid in CIPO must remain high. Mucosal amyloid often has macroscopic abnormalities and abnormal superficial GI biopsies. However, in the case of CIPO, deeper tissue layers for the enteric nervous system and intestinal smooth muscle cannot be assessed on standard endoscopic biopsies. In such cases, surgical full-thickness biopsy, abdominal fat pad, or other solid organ biopsy must be pursued even in the absence of unremarkable endoscopic biopsies. Lastly, we recommend a trial of prucalopride for small bowel dysmotility due to amyloid.

## Conclusions

This case underscores the diagnostic complexity and multidisciplinary effort required in evaluating CIPO, particularly when it is secondary to rare systemic conditions like amyloidosis. In this patient, persistent gastrointestinal symptoms, segmental small bowel dilation without obstruction, and a history of non-ischemic cardiomyopathy prompted a broad differential diagnosis that ultimately led to the identification of amyloidosis. Diagnosis was confirmed by Congo red-positive abdominal fat pad biopsy and cardiac imaging consistent with amyloid infiltration. This case highlights the critical importance of considering infiltrative diseases in unexplained GID and pursuing tissue diagnosis beyond mucosal biopsies when standard evaluations are inconclusive. The therapeutic response to prucalopride in this setting also suggests a potential role for selective 5-HT4 agonists in managing GI amyloid-associated dysmotility. However, longer-term data are needed to evaluate the true efficacy of prucalopride in CIPO from amyloidosis. Early recognition and subtype-specific treatment of amyloidosis are essential to improving outcomes in this challenging and often progressive condition.
